# PROTOCOL: Community‐led total sanitation in rural areas of low‐ and middle‐income countries: a systematic review of evidence on effects and influencing factors

**DOI:** 10.1002/CL2.188

**Published:** 2018-12-20

**Authors:** Josef Novotný, Jiří Hasman, Martin Lepič, Vít Bořil

## 1. Background

### 1.1 The problem, condition or issue

In 2015, an estimated 2.3 billion people did not have access to improved sanitation facilities ensuring a hygienic separation of human excreta from human contact, whereas only 2.9 billion people used safely managed sanitation facilities ensuring a safe disposal and treatment of human excreta in situ or offsite. Of the people without access to improved sanitation facilities, around 892 million practised open defecation (OD), mostly in rural areas of low‐ and middle‐income countries (WHO/UNICEF 2017). Table 1 shows ten countries with the population of more than 10 million which record the highest OD estimates in 2015. It indicates that Sub‐Saharan Africa and South Asia represent the most affected macro‐regions with India being home to more than a half of population practicing OD globally.

**Table 1:** Ten countries with the total population above 10 million with the highest estimates of rural OD rates in 2015

	**Total (thousands)**	**Rural population (%)**	**National OD rate (%)**	**Rural OD rate (%)**
Niger	19899	81	71	85
Chad	14037	78	68	82
Benin	10880	56	55	76
South Sudan	12340	81	61	70
Burkina Faso	18106	70	48	65
Somalia	10787	60	39	60
Angola	25022	56	33	56
India	1311051	67	40	56
Madagascar	24235	65	44	55
Cambodia	15578	79	41	51
WORLD	7349472	46	12	24

Source: WHO/UNICEF (2017)

Prevalent OD is known to have adverse impacts on human health. It contributes to both acute health conditions due to infectious excreta‐related diseases (including the most frequent diarrhoea) and chronic health effects such as anaemia or child stunning (e.g. Mara et al. 2010; Spears et al. 2013; Headey et al. 2015; Mara 2017). In addition to socioeconomic costs associated with adverse health impacts, OD poses a risk of violence against women and other safety concerns (e.g. Mara 2017). Inadequate sanitation is also discussed as a factor of school absence (e.g. Sclar et al. 2017).

The once predominant focus on the construction of toilets has been increasingly supplemented and sometimes replaced by interventions focusing on demand and sanitation behaviour change. The Sustainable Development Goals encompass an ambitious target of eliminating OD worldwide and ensuring access to adequate and equitable sanitation for all by 2030. Sanitation programs have gained momentum and research into sanitation has recently surged. There is a growing need to organise and synthesise gradually accumulated evidence.

### 1.2 The intervention

Community‐led total sanitation (CLTS) has become a prominent behaviour change approach to change sanitation practice at a community level with the main goal to achieve and sustain open defecation free (ODF) status. Since its first implementation in 1999‐2000 in Bangladesh, the CLTS movement has already been used in approximately 60 countries (see www.communityledtotalsanitation.org), some of which have integrated it as part of a national sanitation policy. The introduction of CLTS (and some other related behaviour change approaches to community sanitation) has been presented as a reaction to unsatisfactory result of earlier sanitation interventions focused predominantly on the centralised provision of sanitation hardware and one‐way education about health risks. It might be difficult to find CLTS implementation in a “pure” form in practice as the CLTS implementation is commonly adjusted to local context (e.g. USAID 2018). However, its salient features outlined, for example, in the well‐known Handbook on CLTS by Kar and Chambers (2008) can be summarised as follows:

- Focus on community‐level action and use of social influence factors (e.g. social conformity, social networks, mutual collaboration, solidarity, and surveillance).
- Focus on the introduction of a new social norm around the unacceptability of OD as a key prerequisite for achieving and sustaining the ODF status of a community.
- Use of participatory facilitation towards self‐assessment and community's own decisions rather than direct persuasion through information promotion.
- Use of triggering activities which utilise both positive (e.g. pride) and negative emotions (e.g. disgust and shame) and social motivations to create demand for community action and behaviour change.
- Emphasis on self‐construction of latrines from locally available materials rather than externally provided or subsidised latrines (no hardware subsidies to individual households, no prescribed latrine design).

The CLTS main goal is to eliminate OD and increase latrine coverage and use at a community level. The CLTS implementation consists of the pre‐triggering, triggering, and post‐triggering phases. Pre‐triggering covers planning, training of facilitators, analysing context and identifying risks, and establishing a relationship with local leaders and community. The triggering phase include activities in which facilitators guide the community towards self‐assessment of local sanitation conditions and behaviours, learning about sanitation risks and benefits, changing attitudes, creating demand for collective action, planning the action, and igniting behaviour change. Post‐triggering refers to activities focused on enabling and encouraging the construction of latrines, and a participatory monitoring of progress, including the verification and certification of the community ODF status, subsequent monitoring to sustain the status, and possibly even boost communities' progression up the sanitation ladder. For more information on the CLTS methodology we refer to a handbook by Kar and Chambers (2008) and to a web platform where a larger number of various resources on CLTS can be found: www.communityledtotalsanitation.org.

Some notable examples of other community‐level approaches related to CLTS (see Peal et al. for an overview) represent the Participatory Hygiene and Sanitation Transformation (PHAST), which employs participatory methodology labelled as SARAR (Self‐esteem, Associative strengths, Resourcefulness, Action‐planning and Responsibility) for problem appraisal, action planning, and hygiene and sanitation change at a community level, the Children's Hygiene and Sanitation Training (CHAST), which employs participatory activities adjusted for children, or the Community Health Clubs (CHC), which use a set of regular community meetings to address education about particular health‐related topics. CLTS is primarily focused specifically on the elimination of OD and compared to other approaches it puts more emphasis on social influence factors of behaviour change and negative emotional drivers.

CLTS has been presented as a flexible set of methodological tools that is open to various local adaptations and innovations (Kar and Chambers 2008). Accordingly, the use of CLTS across many developing countries has led to various differences and modifications with respect to the use of particular triggering tools, balance in emphasis on positive versus negative motivational drivers, use of enforcement mechanisms and sanctions, integration of CLTS tools with other approaches, but also regarding fidelity to some of the core elements of CLTS such as no subsidy principle (e.g. Sigler et al. 2015; USAID 2018, p. 8; Venkataramanan et al. 2018; Ficek and Novotný 2018).

The use of negative social influencers such as shaming, stigmatising, and punishing community members in the CLTS implementation has been criticised on ethical grounds and with respect to possible adverse impacts on social equity and intra‐community relations (Bartram et al. 2012, Engel and Susilo 2014, Galvin 2015). These practices also pose a risk for the sense of ownership and sustainability of sanitation change (Bateman and Engel 2018; Novotný et al. 2017). Another concern is that CLTS implementation often leads to poor quality and non‐durable latrines that may, in some instances, exacerbate rather than reduce health risks related to faecal‐oral transmission of pathogens. The evidence on the assumed upgrading of sanitation facilities up the sanitation ladder following the initial step attained through CLTS is limited (e.g. USAID 2018). This is directly related to concerns about the sustainability of sanitation change induced by CLTS so there have been attempts to combine it with other strategies and tools such as sanitation marketing but also with technological support and subsidies for sanitation hardware (e.g. Ficek and Novotný 2018; USAID 2018; Venkataramanan et al. 2018).

### 1.3 How the intervention might work

#### General model of sanitation logic

**Figure 1:**
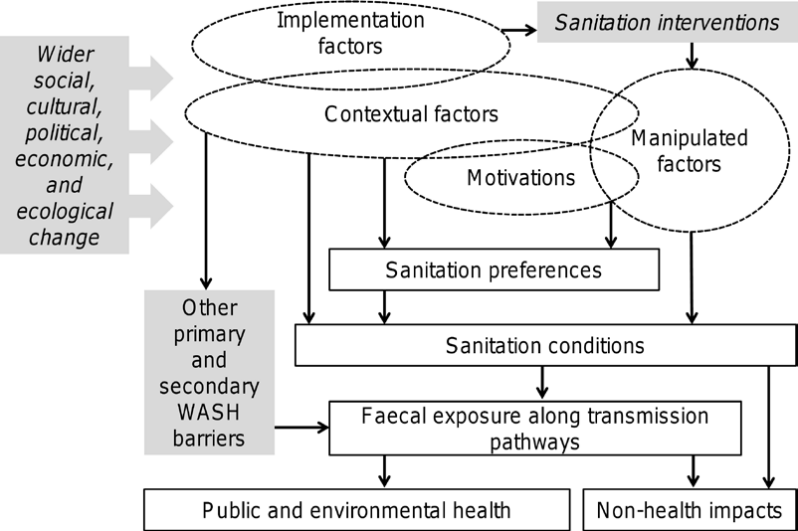
Logic model of sanitation

Before discussing how specifically CLTS might work a logic model of sanitation is outlined in Figure 1 which was adapted from Novotný et al. (2018). It provides a general framework according to which information extracted from particular primary studies will be organised. The explanations and working definitions for the particular components of Figure 1 are as follows:

- The rectangle‐shaped components (except the shaded ones) represent different types of proximate, intermediate and ultimate outcomes. The ellipse‐shaped components represent different types of influencing factors. Whether a variable is classified as outcome or influencing factor depends on its use in an analysed primary study.
- The motivations and contextual factors are predictors that are reported to influence sanitation outcomes independently of a sanitation intervention examined in a given study. By contrast, implementation and manipulated factors operate directly due to the intervention implementation.
- The conceptual distinction between motivations and contextual factors is that the former represents psychosocial (subjective) constructs while the latter refers to various objective characteristics of broadly defined social, economic, political, and natural environment. For example, risk‐perception, attitudes, perceived norms, or awareness about hygienic sanitation practices represent some examples of what we refer to motivations, while factors such as attained education, religion, socioeconomic wealth, or various indicators of natural or policy environments represent contextual factors according to terminology used here.
- By implementation factors we conceive both influences that shape the process of CLTS implementation including deviations from CLTS guidelines and principles.
- Manipulated factors represent parameters directly addressed (i.e. manipulated) by a given intervention. Participation in the intervention itself naturally falls into this category as well as the initial levels of targeted outcomes such as baseline OD or latrine coverage rates. In addition, manipulated factors are also those sanitation drivers that the intervention targets according to its theory of change (i.e. variables that causally link inputs with outcomes). As CLTS represents a behaviour change approach, psychosocial parameters such as the perception of social norms or various attitudinal variables may represent relevant examples of manipulated factors. Therefore, efforts to identify manipulated factors in analysed literature means effort to map the theory of change.
- Overlaps between particular types of factors indicated in Figure 1 symbolise the already mentioned fact that the classification depends on how they are interpreted in a given study. For example, the history of an earlier subsidy‐based sanitation intervention in a targeted community is a contextual factor that may influence sanitation preferences but also the quality of implementation of subsequent intervention so it becomes implementation factor too. Similarly, preference for OD represents a known motivational driver, which becomes a manipulated factor if directly addressed by a considered intervention.
- Sanitation conditions contain objective outcome indicators characterising sanitation situation such as indices of latrine coverage and use.

Sanitation preferences refer to outcome variables that are subjective psychosocial constructs such as willingness to adopt or pay for a latrine or other types of behavioural intentions related to sanitation. These are choices that logically precede sanitation conditions in the causal model.

#### How might the CLTS intervention work?

The CLTS has been built around a few core principles and a set of implementation guidelines described in the CLTS Handbook (Kar and Chambers 2008). Rather than being derived from a theory, CLTS has been a participatory methodology that evolved in practice, based on real‐life situations, from sharing and discussing experiences with participants in communities (Chambers 2012). CLTS thus does not draw on a single behaviour change theory, though its particular aspects can be related to multiple behaviour change frameworks (Sigler et al. 2015). Although the logic of CLTS might seem intuitive from the guidelines, comprehending its underlying mechanisms is not an easy task. Main inputs, activities, and outcomes of CLTS are known. However, causal pathways between them have not been explicitly operationalised and main focus factors manipulated by CLTS have not been explicitly specified. As Harter et al. (2018, p. 1) put it: “little is known on how CLTS works.”

Table 2 may be indicative in this respect. It lists factors and characteristics to be addressed in the CLTS implementation according to the CLTS Handbook (Kar and Chambers 2008). The list clearly reflects the community based nature of CLTS and encompasses mainly psychosocial drivers. This exercise also documents that negative motivational influencers and social pressures should mainly be used in the triggering phase, whereas more positive forces associated with facilitating collaborative action or solidarity in a community are more common for the post‐triggering phase. However, the CLTS Handbook first of all provides a practically oriented discussion of implementation activities and it doesn't contain an explicit theory of change. Also in a majority of other materials on CLTS, the theory of change (if any) tends to be only loosely defined.

**Table 2:** Instrumental factors and characteristics to be addressed according to the CLTS Handbook

	Specific to particular CLTS phases		
General	Pre‐triggering	Triggering	Post‐triggering
Community empowerment; social awakening; self‐learning; realisation of collective benefits; collective action	Trust ‐ relationship with community and natural leaders	Collective sense of disgust and shame; embarrassment; sense of un‐cleanliness and impurity; inconvenience; social pressures due to facilitated tensions in community (e.g. in dirtiest neighbourhood or shit calculation exercises); realisation of economic costs; collective efficacy to take action	Collaborative action planning; cooperation and social solidarity; identifying and linking hardware suppliers; supporting natural leaders; encouragement; pride; competition between communities; participatory monitoring; social surveillance

General conceptual models for behaviour change in Water, Sanitation, and Hygiene (WASH) may be helpful to hypothesise about factors potentially manipulated by CLTS. The Risks, Attitudes, Norms, Abilities, and Self‐regulation (RANAS) model developed by Mosler (2012) is one of them, which distinguishes five blocks of psychosocial determinants of WASH behaviour. The CLTS approach is comprehensive in the sense that each of these RANAS factor blocks can possibly be addressed by some of the CLTS elements. In terms of presumed causality, the RANAS factors logically follow rather than precede factors and characteristics extracted from the CLTS Handbook (Table 2). Conceptually, the RANAS factors represent the intermediate components of causal chain that interlink the characteristics in Table 2 with expected short‐term outcomes denoted as sanitation preferences in Figure 1. As such, a basic skeleton of hypothesised causal chain for CLTS can be represented as: *CLTS intervention → Factors and characteristics in* Table 2 *→ RANAS factor blocks → Sanitation preferences → Sanitation conditions → Health and non‐health impacts of improved sanitation conditions.*

Sanitation interventions involve complex human‐environment interactions which predetermine the implementation and results to be considerably sensitive to specifics of natural and social environments. This is well reflected in the CLTS guidelines which list various favourable and challenging conditions and emphasise the flexibility of CLTS methodology and a need to adjust it based on local context. Accordingly, factual emphasis on specific behaviour change drivers may vary between programmes and organisations implementing CLTS.

In spite of this and also despite the abovementioned CLTS comprehensiveness, the use of emotional drivers in the triggering phase, particularly negative ones, is often viewed as a distinctive CLTS feature. It is to activate social/peer pressures and establishing a new community norm of unacceptability of OD. From this point of view, the perception of social norms around OD and latrine use represents a key manipulated factor that distinguishes CLTS from other behaviour change approaches. However, the understanding of how different types of normative pressures (e.g. descriptive versus injunctive norms) addressed in CLTS operate is still in the infancy (e.g. Dooley et al. 2016; Novotný et al. 2017).

The basic causal chain above can be compared to the theory of change developed for a systematic review of approaches to promote handwashing and sanitation behaviour (de Buck et al. 2016). That model recalls different elements of behaviour change in terms of intention, use, and habit. With the terminology established above, behavioural sanitation intentions represent sanitation preferences while the measures of use represent sanitation conditions, whether the sanitation behaviours are routinised or based on some cognitive effort.

The theory of change developed in De Buck et al. (2016) additionally elaborates on implementation factors, while distinguishing between various types of programme environment factors and recipient‐related factors. This is relevant here because a notable share of literature on CLTS focuses on the process of CLTS implementation. In this respect, Venkataramanan et al. (2018) identified ten different types of factors related to the quality of CLTS implementation. They can be divided into factors that describe the quality of implementation process (e.g. triggering quality and quality of facilitators, frequency and effectiveness of follow‐up visits, planning etc.) and those related to the use of specific implementation tools (e.g. provision of incentives to community volunteers, rewards for attaining ODF status, use of enforcements measures) or deviations from the core CLTS principles (e.g. use of subsidies or technical support for latrine construction).

In addition to the factors related to the processes of CLTS implementation, Venkataramanan et al. (2018) identified the following types of CLTS influencers: (a) policy environment (e.g. political will, ambitious targets, history of previous interventions or other ongoing interventions), (b) administrative context (e.g. coordination between implementing organisations, local government ownership of CLTS, financial arrangements), (c) community environment (e.g. various climate and physical environment characteristics), (d) community capacity (access to materials for toilet construction, financial resources, and technical knowledge), (e) community participation (e.g. village level leadership, social cohesion, sense of responsibility), (f) community behaviour (e.g. expectations of subsidies, preference for OD, preference for better latrine, traditional beliefs regarding OD or latrine use, other priorities than sanitation). With the present terminology as in Figure 1 above, these influencers correspond to various types of contextual factors and motivations.

### 1.4 Why it is important to do the review

Since its inception in 1999‐2000, the CLTS approach has quickly become widely used. It has gained a reputation for being an inexpensive, simple, participatory, community‐empowering, and effective strategy for the elimination of open defecation (OD). The potential of CLTS to improve human health and the sustainability of outcomes achieved through CLTS are nevertheless debated (e.g. Pickering et al. 2015; Crocker et al. 2017a; Venkataramanan et al. (2018; USAID 2018) and there is thus an obvious need to synthesise evidence on the CLTS effectiveness as well as influencing factors.

Prior to this review, we conducted an interview survey among practitioners experienced with CLTS implementation in 14 different countries (Ficek and Novotny 2018). We found a contrast between the predominantly positive assessments, popularity, and massive use of CLTS in practice, on the one hand, and the so far very limited evidence of its longer‐term impacts both in literature and among practitioners themselves, on the other hand. At the same time, we realised that the evidence synthesis of the CLTS effectiveness would most likely be insufficient to inform policy and practice because of contextual heterogeneity and adjustments. A systematic review of evidence on which factors influence CLTS outcomes and how thus obviously has a practical appeal.

There are a few recent systematic reviews published since 2016 in the area of sanitation (de Buck et al. 2017; Novotny et al. 2018; Garn et al. 2017; Sclar et al. 2016; Freeman at al. 2017; Sclar et al. 2017) which did not focus specifically on the evidence on CLTS approach. When we started our work on this protocol, we were not aware of any systematic review concerned specifically with either the results of CLTS approaches or factors which affect the use of CLTS approaches.

However, a mixed methods systematic review of evidence on CLTS was recently published (Venkataramanan et al. 2018; published on 2 Feb, 2018) and another review study (though not a systematic review) on various aspects of CLTS emerged in April 2018 (USAID 2018). Although the systematic review by Venkataramanan et al. (2018) addressed very similar research questions as our review, its approach is different as its major goal was to assess the quality of available evidence on CLTS as:

- A few interesting primary studies were published in the last year including some rigorous impact evaluations that were not covered in Venkataramanan et al. so the evidence is growing.
- Venkataraman et al. (2018, p.2) mention in their work that: “In the case of multiple documents reporting data and findings from the same intervention or study, only the most recent document was included.” We already found out that for several interventions more than one study exists and that these studies often complement rather than duplicate one another. We will attempt to maximise the information on an intervention by extracting relevant information on the same intervention from multiple studies whenever applicable. We expect that this will be important with respect to evidence on the second review question.
- Venkataramanan et al. (2018) focused on all identified studies on CLTS because one of their main objectives was to assess the quality of existing evidence. However, it also means that they extracted factors influencing CLTS from all of the 200 studies of which a large part was assessed as of low quality. We consider their finding about generally poor quality of evidence as a starting point and, unlike Venkataramanan et al. (2018), we will use a quality and information content appraisal to select and consider only studies of acceptable quality for the extraction of data on the second review question.
- Venkataramanan et al. (2018) descriptively characterised outcomes and influencing factors identified from the considered primary studies but did not report on links between particular factors and specific outcomes. The latter is one of the main goals of our review with respect to the second review question. In other words, we will not only analyse which factors and outcomes are reported in literature but also how important are particular factors with respect to specific outcomes.
- Unlike Venkataramanan et al. (2018) we will focus only on studies that analysed CLTS in rural or semi‐rural settings. We believe that sanitation poses quite different challenges in urban settings and that it is not appropriate to synthesise evidence from urban and rural settings together.

The findings of our systematic review will inform sanitation practitioners and researchers about the existing evidence on the effectiveness of CLTS approach. Beyond this, our review will help practitioners and researchers to identify the key factors that can be consequential with respect to the implementation and results of CLTS interventions as synthesised from primary studies of acceptable quality.

## 2. Objectives

The systematic review will address the following review questions:

1) What are the effects of CLTS approach in rural areas of low‐ and middle‐income countries on the measures of sanitation conditions?
2) Which factors influence the implementation and results of CLTS approaches in rural areas of low‐ and middle‐income countries and how?

The first research question will primarily focus on the effects of CLTS on the measures of sanitation conditions. These include measures of OD, latrine use, latrine ownership, latrine quality, and ODF status of community. Effects of CLTS on sanitation preferences (as more proximate outcomes in causal chain) or more distant health and non‐health benefits of improved sanitation conditions will also be recorded but will not represent main parameters with respect to the first research question.

Sanitation interventions revolve around complex human‐environment interactions, target multiple proximate and ultimate outcomes at different levels (e.g. individual versus community level), and they are highly sensitive to initial conditions and various specifics of social and natural environment. They can thus be thought of as interventions in “complex systems” (Novotný et al. 2018) which makes any quantitative synthesis of intervention effects difficult. As such, we consider both of the review questions proposed above as similarly important. We believe that generalisability of research findings in the area of sanitation is necessarily indirect and the focus on intervention effectiveness must be done together with a systematic focus on the role of contextual factors.

The two research questions will be accomplished through the following steps. First, we will systematically identify studies that analyse CLTS approaches and report on their results. Second, we will appraise these studies along the predefined criteria developed for the purposes of this review and determine the final sample of studies. Third, we will identify sanitation outcomes analysed in the final sample of studies and typologically classify these outcomes. In addition to a thematic classification, we will distinguish between the proximate and ultimate outcomes as indicated in the logic model of sanitation in Figure 1 and assess whether and how the inter‐linkages between intervention and these two types of outcomes are established in particular studies. Fourth, we will assess the evidence on effectiveness of CLTS with respect to different types of outcomes. Fifth, we will identify factors influencing CLTS implementation and reported sanitation outcomes and typologically classify these factors and outcomes with respect to both the thematic focus and their typology indicated in Figure 1. Sixth, we will examine and characterise how the theory of change behind CLTS is represented. Seventh, we will attempt to assess how different contextual and implementation factors (Figure 1) affect the results of CLTS.

## 3. Methodology

The methodology proposed below corresponds to the Campbell Collaboration Guidelines on Systematic Review Methods.

### 3.1 Criteria for including and excluding studies

#### Types of study designs

For the first review question (i.e. “What are the effects of CLTS”…) only quantitative counterfactual studies with prospective control design that report on results of CLTS interventions or their direct modalities will be considered. Therefore, the eligible studies should include a control or comparison group and they may have randomised (whether individually‐ or cluster‐randomised), quasi‐randomised, or non‐randomised design. ‘Before‐after’ studies with a comparison group will be included. ‘Before‐after’ studies which include control variables but which do not include a non‐intervention comparison group will not be included in synthesis of findings for primary outcomes. However, they will be recorded and descriptively characterised. Although these studies do not allow causal attribution of positive effects, they may still provide interesting findings on negative (adverse) results of CLTS with important practical implications regarding highly discussed issues around CLTS sustainability.

For the second review question (i.e. “Which factors influence the implementation and results of CLTS and how”…) no a priori restrictions with respect to study design will be applied. However, only those primary studies that report on the results of CLTS will be considered and only those studies that will meet the quality and information content criteria described below will be considered for data extraction. It means that studies which, for example, examine the process of CLTS implementation without reporting on its results will be excluded. Quantitative experimental, quasi‐experimental, and observational studies which will satisfy our appraisal criteria (described below) will be considered. Qualitative studies that analyse relationship(s) between CLTS and proximate or ultimate sanitation outcomes will also be considered.

#### Types of participants

No restrictions on demographic characteristics of participants will be applied. Based on the logic model of sanitation in Figure 1, the defining parameters of participants (either individuals or communities) ‐ whether demographic, socioeconomic, sociocultural, ecological ‐ will represent contextual factors. We expect that in most studies individuals or households will be the basic units of observation, though they may simultaneously report community‐, village‐, or region‐level contextual characteristics. Studies which consider entire communities, villages, or higher‐level entities (e.g. districts) as the basic units of observation will also be identified but considered as a separate category.

#### Types of settings

The review will focus on evidence on the CLTS use in rural or semi‐rural areas of low‐ and middle‐income countries (World Bank classification). Studies focusing on urban or peri‐urban settings will be excluded. The definitions of rural and semi‐rural settings used in particular studies will be considered.

#### Types of interventions

The review will examine evidence on primary studies reporting on the results of CLTS approach including its direct modalities. To be included, a study should either acknowledge that it addresses a CLTS or explicitly establish a clear link to CLTS methodology. The latter means the focus on behaviour change to reduce OD at community level where the use of CLTS participatory techniques described above represents an important element. If the link to CLTS is not made explicit, the study will not be considered for this review. Therefore, studies on other behaviour change approaches such as PHAST, SARAR, CHAST, CHC (see Section 1.2) will not be included unless they explicitly specify that these approaches are combined with CLTS.

However, we will include studies examining the direct modalities of CLTS and its combinations with other approaches. To mention some notable examples, it applies for the use of CLTS in Ethiopian Community‐Led Total Sanitation and Hygiene (CLTSH), in UNICEF's Community Approaches to Total Sanitation (CATS), or Indonesian Total Sanitation and Sanitation Marketing programme (TSSM). Primary studies focusing on results of Indian Total Sanitation Campaign (TSC) will only be considered if the use of CLTS techniques in a given case study is explicitly acknowledged.

We learned from the survey among CLTS practitioners (Ficek and Novotny 2018) that the CLTS approach has often been adjusted to local context. We thus don't expect that all of the salient features of CLTS listed above will always clearly be identifiable from scrutinised primary studies. The mapping of variations in the considered primary studies with respect to their underlying theory of change will be one part of this review. Combinations of CLTS with other approaches as well as specific adjustments of CLTS will be recorded and analysed as implementation factors.

#### Comparison

Non‐intervention settings, other sanitation approaches than CLTS, or various modalities of the CLTS approach may be considered as comparators.

#### Types of outcome measures

Multiple different outcomes are often analysed in primary studied on CLTS, as documented in Table 4 of a recent systematic review by Venkataramanan et al. (2018). The logic model of sanitation outlined in Section 1.3 conceptually distinguished between proximate outcomes describing sanitation preferences and conditions and ultimate outcomes in terms of health and non‐health effects on well‐being (the distinction between proximate and ultimate outcomes thus corresponds to that between primary and secondary outcomes).

With respect to the first research question we will only consider studies which report on outcomes characterising changes in sanitation conditions within a given time‐frame. This typically includes the measures of OD, latrine use, latrine ownership or access, latrine quality, and ODF status of community. Identification of outcomes related to sanitation preferences that logically precede sanitation conditions in the causal chain as well as more ultimate outcomes such as indices of faecal exposure and health and non‐health benefits will be done for the second research question but not used for meta‐analysis. The mapping of outcomes will also concern reported unintended (including adverse) effects. As for other outcomes, we will seek to account for factors that influence the occurrence of the unintended effects. The consideration of possible adverse effects can be important because of already mentioned concerns about unethical practices and other adverse impacts of CLTS.

#### Duration of follow‐up

We will not apply any a priori restriction with respect to the duration of follow‐up. If multiple follow‐ups incorporated, the maximum follow up length will be considered.

### 3.2 Search strategy

**Figure 2:**
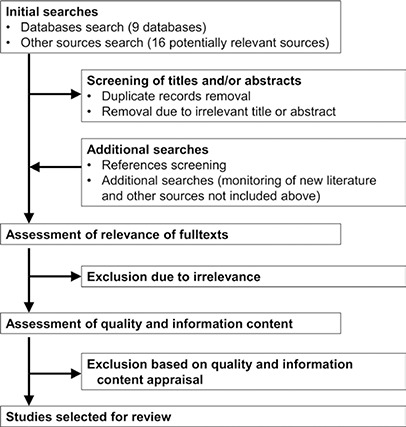
Flow diagram of study search and selection

The flow diagram in Figure 2 outlines the process of study search and study selection. Both peer‐reviewed studies in journals and grey literature will be searched as previous reviews corroborated the importance of considering grey literature (Novotný et al. 2018; Venkataramanan et al. 2018). We will search literature published since 1999 as the CLTS approach was firstly used in 1999‐2000 (Kar and Chambers 2008). Due to capacity constraints we will not specifically search for non‐English documents. This will be described among limitations of the present review. If our automatic searches yield potentially relevant studies written in other languages than English, we will consider them.

The list of searched databases including the search strings is shown in Table 2. In addition, the following 16 potentially relevant sources will be searched either automatically (if a search option available) or manually: Water and Sanitation Program library, Practical Action, India Sanitation Portal, Community‐Led Total Sanitation (www.communityledtotalsanitation.org), Sanitation Marketing, 3ie International Initiative for Impact Evaluation, IRC (www.ircwash.org), J‐PAL (www.povertyactionlab.org), WaterAid, UNICEF, WaterSHED, Plan International, Department for International Development (DFID), Sanitation Updates (https://sanitationupdates.wordpress.com), Water, Engineering and Development Centre at Loughborough University, Institute of Development Studies at the University of Sussex.

References in the relevant studies will be screened for other potentially relevant primary studies and we will also monitor new literature published after our systematic searches. The authors of primary studies not available or with missing parts will be contacted through email with requests to provide the missing information.

Importantly, we will check our search results against the set of 200 primary studies included into the recent systematic review of CLTS by Venkataramanan et al. (2018).

Search of databases will be repeated before determining the final sample of studies to include most recent studies as possible.

**Table 2:** Search databases and search strings

**Database**	**String**
*ProQuest Central*	all (CLTS OR ((sanitation OR latrine OR toilet OR privy OR disposal) AND community AND intervention))
*Science Direct*	CLTS OR ((sanitation OR latrine OR toilet OR privy OR disposal) AND community)
*Web of Science*	CLTS OR ((sanitation OR latrine OR toilet OR privy OR disposal) AND community)
*Scopus*	CLTS OR ((sanitation OR latrine OR toilet OR privy OR disposal) AND community)
*PubMed*	CLTS OR ((sanitation OR latrine OR toilet OR privy OR disposal) AND community)
*The British Library*	CLTS OR ((sanitation OR latrine OR toilet OR privy OR disposal) AND community)
*Google Scholar*	allintitle: CLTS OR “total sanitation”
*World Bank Publications*	CLTS OR ((sanitation OR latrine OR toilet OR privy OR disposal) AND community)
*WHO ‐ Iris*	CLTS OR ((sanitation OR latrine OR toilet OR privy OR disposal) AND community)

### 3.3 Description of methods used in primary research

The recent systematic review of CLTS by Venkataramanan et al. (2018) can indicate the nature of available primary studies. Of 200 primary studies considered in that review, 14 were quantitative impact evaluations (experimental, quasi‐experimental, or before‐after comparisons), 29 were qualitative studies, and the remaining 157 were case studies (mixed methods, cross‐sectional, project reports etc.). However, as already explained above, an important goal of Venkataramanan et al. (2018) was to examine the quality of evidence on CLTS so they considered all available primary studies (N=200). By contrast, we expect that our final sample of primary studies will be considerably less extensive because we will only consider those studies that will meet the pre‐defined quality and information content appraisal criteria. Moreover, Venkataramanan et al. (2018) also included studies that focus on the process of CLTS implementation and do not report on its results (this applies for several qualitative studies and many case studies). These studies will not be included in our review.

#### Examples of primary studies

We can refer to the following “family” of four articles on CLTS in Ethiopia and Ghana (Crocker et al. 2016a; 2016b; 2017a; 2017b):

- Crocker et al. (2016a) is a quasi‐experimental study which compared the results of CLTS facilitated by teachers in 111 non‐randomly selected villages in two regions of Ethiopia with the results of CLTS facilitated by health workers and local leaders (as conventional in Ethiopia) in 54 villages in the same regions. The quantitative part of analysis used difference‐in‐differences technique and focused on changes in self‐reported OD rates and private latrine use (primary outcomes) in the 12 months time frame. Some other outcomes related to latrine quality and handwashing were also measured. The study compared two variations of CLTS but didn't consider no‐intervention control group.
- In their other study, Crocker et al. (2016b) report results of a cluster‐randomised trial which analysed the impacts of adding natural leaders training to a CLTS intervention in three dissimilar regions of Ghana. After 5 months of CLTS implementation in 60 villages, natural leaders were identified and trained in a half of randomly selected villages. The implementation then continued for next 12 months (which was the time frame of this study) and the study compared results of CLTS attained in villages with and without natural leaders training. The focal outcomes were changes in self‐reported OD, private latrine ownership and use, and latrine quality.
- Crocker et al. (2017a) considered the same interventions in Ethiopia and Ghana as in the studies referred to above (i.e. Crocker et al. 2016a and 2016b) but extended the time frame by re‐surveying 3831 households one year after the end of interventions. The main goal was to evaluate the sustainability of effects of the CLTS interventions on latrine use and quality.
- The fourth study by Crocker et al. (2017b) once again considered the same interventions in Ethiopia and Ghana but reported on the costs of their implementation disaggregated by various parameters rather than on their results.

The first three studies (i.e. Crocker et al. 2016a, 2016b, and 2017a) report CLTS results but the fourth study (Crocker et al. 2017b) does not so this latter article will be excluded. The third study (Crocker et al. 2017a) presents additional results for the interventions already analysed in the first two papers (Crocker et al. 2016a, 2016b). Our goal will be to maximise evidence on specific interventions but prevent a double‐counting. Additional information from this study (Crocker et al. 2017a) will thus be appended to that from the first two studies. We also observed that none of the examples above considers non‐intervention comparison group; they use ‘active’ comparators. All these published articles provide interesting comparative evidence on CLTS in two different countries and dissimilar regions within the countries. The notable finding is the confirmation of a high importance of local contexts. This is explicitly emphasised in one of the studies: “The impact of CLTS and subsequent sustained latrine use varied more by region than by intervention, indicating that context may be as or more important than the implementation approach in determining effectiveness” (Crocker et al. 2017a, p. 555). It once again indicates the importance of various context‐related heterogeneities which challenge any attempt for quantitative characterisation of pooled effect(s).

### 3.4 Criteria for determination of independent findings

If multiple documents report findings from the same study (same data set), we will consider all documents in order to maximise information on the examined review questions, while avoiding inaccuracies related to double‐counting of participants.

### 3.5 Details of study coding categories

#### Quality and information content appraisal

For the first review question on the CLTS effectiveness, no study will be excluded on the basis of quality assessment alone. The risk of bias of eligible studies will be assessed on a 4‐point scale (high, medium, low, and uncertain) along criteria 6, 7 (related to external validity) and 13‐15 (related to internal validity) in Table 3.

**Table 3:** Appraisal criteria and their justification

**Criterion**	**Justification**
*1. Publication type*	Type of publication uses to be connected to the quality of peer‐review assessment which can have an impact on the overall quality of publication. In our previous work we showed that articles published in peer‐reviewed journals indexed at Web of Science database reveal on average better quality than articles in other peer‐reviewed journals and grey literature (Novotný et al. 2018, Suppl. material A, Figure S2b).
*2. Source of data*	The study should use primary data. A combination of data from more sources is preferred.
*3. Size of the study sample*	The larger samples should provide stronger evidence of results. The size of the study sample should be justified.
*4. Generalisability of results*	The study should be held in sufficient number of communities which should differ in several aspects in order to ensure at least some external validity of results.
*5. Sustainability of results*	The study should be held with sufficient time since the intervention to enable to evaluate the sustainability of the results.
*6. Selection of communities*	The studied communities should be selected by rigorous manner in order results to be representative. The selection process has to be well described.
*7. Sampling of respondents*	The respondents/households should be selected by rigorous manner in order results to be representative. The selection process has to be well described.
*8. Description of research process*	The whole process of how the research was conducted should be described in detail. Definition of variables presented in the study should be presented.
*9. Explanation of intervention*	The logic of the studied interventions and its reasoning should be well described. The beneficiaries of the intervention should be selected based on the clear and appropriate criteria.
*10. Conclusions*	It should be clear on which data are the conclusions based. These data should be processed adequately.
*11. Characteristics of communities*	The study should take into account as much characteristics of the studied communities that may affect the sanitation outcome as possible.
*12. Characteristics of respondents*	The study should take into account as much characteristics of the respondents or households that may affect the sanitation outcome as possible.
*13. Selection of comparison group*	The study should present data for comparison group in order to rigorously extract the effect of intervention.
*14. Quality of comparison group data*	Comparability of both intervention and control group should be tested and possible differences should be rigorously dealt with. Attrition between *before* and *after* data is acceptable. *Before* data are prospective.
*15. Allocation to intervention*	The randomisation method should be appropriate and clearly described. Random and possible non‐random components of sequence generation process and selection of participants should be appropriate and clearly described.
*15. Measuring effect of intervention*	Effect of intervention should rigorously be measured

For the second review question on factors affecting the results of CLTS, only those studies that will meet our predefined criteria of quality and information content will be further used for data extraction. Here, we will use a similar approach as in our previous review (Novotný et al. 2018) adjusted with regards to the second research question of this review. It was inspired by EPPI‐Centre (2010), which suggests considering three dimensions including the methodological quality, methodological relevance and topic relevance of reviewed studies. For this review, we excluded criteria addressing methodological relevance as we decided to apply no restrictions on methods. No predefined checklist was found appropriate with respect to the scope of this review so we decided to create an own list of 12 criteria listed in Table 3. The selection was mainly informed by checklists published in Petticrew and Roberts (2006).

We will use a four‐point grading system for each of the criteria on a scale from 0 (totally unsatisfactory) to 3 (fully achieves a criterion) similarly as in e.g. Khan et al. (2003) or Hulland et al. (2015). The aggregate scores for individual studies will be calculated as the unweighted means of the grades obtained for all relevant criteria. It means that criteria irrelevant for a given study because of its design (e.g. allocation to intervention for observational primary studies) will not be considered in the calculation of aggregate score.

The studies will be divided into the four categories of low quality and information content evidence (scores below 1.50), average (scores between 1.50 and 1.99), above‐average (scores between 2.00 and 2.49), and high quality and information content (scores of 2.50 or more). Only studies classified as above‐average or high quality will be used for data extraction.

Although the aggregate score will be used, we will examine how particular appraisal criteria affect the final selection of studies and examine the robustness of results with respect to “border‐line” decisions about exclusion/inclusion of individual studies along particular criteria. We will also explore the key sources of study quality and consider presenting results stratified according to selected study quality components.

The appraisal will be conducted by two reviewers. They will firstly assess the same pairs of studies randomly selected from the analysed sample and then compare and discuss their assessments to eliminate differences in understandings to particular criteria. Subsequently, they will work independently but disputable cases will be discussed and resolved together.

The quality appraisal of individual studies along particular criteria listed above will be recorded into pre‐defined form. Where judgments are based on assumptions made by considering information taken from not publicly available documents, this will be stated in comments in the quality appraisal form. The form (assessment of individual studies) will be made available together with review.

#### Data extraction

Basic descriptive characteristics of particular studies will be extracted including the description of intervention, date, information on design, characteristics of sample and sample size, time frame and other relevant characteristics (much of the information will be also needed during the quality and information content assessment).

For the first review question, we will extract results on effect sizes (mean difference or standardised mean difference (SMD) for continuous outcomes and odds ratios (OR) for dichotomous outcomes) or information needed for their calculation and check that effect sizes are calculated consistently. If necessary information, for example for calculation of SMD and its standard error, is not reported we will seek the information from the authors through email. For cluster‐allocated studies we will check whether a possible unit of analysis error was addressed and if not we will use a unit of analysis correction.

With respect to the extraction of material for the analysis of the second review question, the basic unit of observation will correspond to pairwise relationships between outcomes and their influencing factors. We will consider all such relationships identified in the final sample of studies or their supplementary materials (or errata), whether established quantitatively or qualitatively. For each relationship, we will record the descriptions of explanatory variable (influencing factor) and sanitation outcome and the nature of the relationship together with the effect size, if applicable.

The data extraction will be done by three reviewers. Afterwards, the lead author will once again scrutinise the full texts of all analysed primary studies in order to check whether all relevant findings were correctly extracted.

### 3.6 Statistical and other procedures and conventions (including the treatment of qualitative research)

Feasibility of meta‐analysis will be considered to examine the first research question on CLTS effectiveness. Meta‐analysis will be used only if two or more effect sizes are available for sufficiently similar outcome construct, comparison group, and intervention type. As already stated above, measures of OD, latrine use, latrine ownership or access, or ODF status of community will be meaningful outcomes. Non‐intervention setting or other intervention may be considered as control conditions. If meta‐analysis will not be found feasible, we will characterise results of identified studies descriptively including their similarities and dissimilarities. We will also still try to use forest plot(s) to present findings. Experimental and non‐experimental studies will be treated separately in the attempts to quantitatively synthesise results on CLTS effectiveness. We will check for accuracy of numeric data by comparing magnitudes and directions of effects reported by individual studies and further exploring possible reasons of discordances (if any detected). If a study is included with more than two intervention arms, only intervention and control groups that meet the eligibility criteria will be considered. A random‐effects meta‐analysis will be used to synthesise effects across studies, by outcome measure. Studies will be included in forest plots and meta‐analysis regardless of whether they report statistically significant findings. Influence of clustering and non‐standard design features of individual studies on results will be considered in sensitivity analysis.

To examine our second review question (Which factors influence the implementation and results of CLTS and how?) we will proceed as follows. First, we will thematically classify outcomes and influencing factors. Second, we will typologically classify particular factors, outcomes, and relationships into the components of the logic model of sanitation in Figure 1. Using this “decomposition” we will try to map an underlying theory of change and assess variations between particular studies. Third, we will assess which factors influence which sanitation outcomes and how. In addition to a descriptive characterisation of the relationships between different types of factors and outcomes, we will also attempt for a graphical representation of the patterns in these relationships, for example using a network visualisation of ‘sanitation nexus’ as in Novotný et al. (2018). The procedure is applicable to both quantitative and qualitative evidence.

### 3.7 Data collection forms

In the literature screening phase, we will use a predefined form with the following columns: study identification (ID); reviewer; study citation; whether focuses on effects of sanitation intervention; whether combined with other interventions or WASH intervention components; type of intervention; country; region/location; date of intervention; sample size and character; settings (rural/urban/specify); main outcome(s); whether reports on influencing factors; usability with respect to review questions; other comments.

In the data extraction phase we will use a data extraction form for recording relevant information from the final sample of primary studies. It will be organised according to the components of Figure 1 (logic model of sanitation). It will contain four separate sheets for recording information on (i) outcomes, (ii) implementation factors, (iii) manipulated factors, and (iv.) contextual factors into separate lined for each of the studies. The sheet for recording outcomes will distinguish between different types of proximate outcomes and different types of ultimate outcomes (faecal contamination along transmission pathways, health‐related outcomes, non‐health outcomes, other outcomes). The sheet for implementation factors will record information on intervention (several parameters on design and implementation, incl. info on follow up period, information about departures from core CLTS principles etc.) and several types of factors that may directly affect implementation (environmental, political, social, history of previous interventions). The sheet for manipulated factors will record initial conditions of main outcomes and factors that are explicitly considered as manipulated according to the theory of change of a given intervention (if indicated in a primary study or other its supporting materials). The composition of the sheet on contextual factors will be informed by Novotny et al. (2018) and it will record several broader types of contextual factors (socioeconomic, demographic, spatial and environmental, sociocultural, demographic, psychosocial, and other).

## Review authors

**Lead review author:** The lead author is the person who develops and co‐ordinates the review team, discusses and assigns roles for individual members of the review team, liaises with the editorial base and takes responsibility for the on‐going updates of the review.

**Table:** 

Name:	Josef Novotny
Title:	Dr.
Affiliation:	Charles University, Faculty of Science
Address:	Albertov 6
City, State, Province or County:	Prague
Post code:	12843
Country:	Czechia
Phone:	+420605469487
Email:	pepino@natur.cuni.cz
**Co‐authors:**	
Name:	Jiří Hasman
Title:	Dr.
Affiliation:	Charles University, Faculty of Science
Address:	Albertov 6
City, State, Province or County:	Prague
Post code:	12843
Country:	Czechia
Phone:	
Email:	jiri.hasman@natur.cuni.cz
Name:	Martin Lepič
Title:	Mr.
Affiliation:	Charles University, Faculty of Science
Address:	Albertov 6
City, State, Province or County:	Prague
Post code:	12843
Country:	Czechia
Email:	martin.lepic@natur.cuni.cz
Name:	Vít Bořil
Title:	Mr.
Affiliation:	Charles University, Faculty of Science
Address:	Albertov 6
City, State, Province or County:	Prague
Post code:	12843
Country:	Czechia
Email:	vit.boril@natur.cuni.cz

## Roles and responsibilities

Give a brief description of content and methodological expertise within the review team. It is recommended to have at least one person on the review team who has content expertise, at least one person who has methodological expertise and at least one person who has statistical expertise. It is also recommended to have one person with information retrieval expertise.

Please note that this is the *recommended optimal* review team composition.

- Content: Josef Novotny
- Systematic review methods: Jiri Hasman
- Statistical analysis: Jiri Hasman, Josef Novotny
- Information retrieval: Jiri Hasman, Martin Lepic, Vit Boril

## Sources of support

Material support has been provided from the Department of Social Geography and Regional Development, Faculty of Science, Charles University, Prague, Czechia. We have secured a specific funding for the period 2015‐2017 from the Czech Science Agency. We will seek more funding for 2018 onwards.

## Declarations of interest

The authors are not aware of any potential conflict of interest.

## Preliminary timeframe

Approximate date for submission of the systematic review is by the end of 2018.

## Plans for updating the review

To be determined.

## AUTHOR DECLARATION

### Authors' responsibilities

By completing this form, you accept responsibility for preparing, maintaining and updating the review in accordance with Campbell Collaboration policy. The Campbell Collaboration will provide as much support as possible to assist with the preparation of the review.

A draft review must be submitted to the relevant Coordinating Group within two years of protocol publication. If drafts are not submitted before the agreed deadlines, or if we are unable to contact you for an extended period, the relevant Coordinating Group has the right to de‐register the title or transfer the title to alternative authors. The Coordinating Group also has the right to de‐register or transfer the title if it does not meet the standards of the Coordinating Group and/or the Campbell Collaboration.

You accept responsibility for maintaining the review in light of new evidence, comments and criticisms, and other developments, and updating the review at least once every five years, or, if requested, transferring responsibility for maintaining the review to others as agreed with the Coordinating Group.

### Publication in the Campbell Library

The support of the Coordinating Group in preparing your review is conditional upon your agreement to publish the protocol, finished review, and subsequent updates in the Campbell Library. The Campbell Collaboration places no restrictions on publication of the findings of a Campbell systematic review in a more abbreviated form as a journal article either before or after the publication of the monograph version in Campbell Systematic Reviews. Some journals, however, have restrictions that preclude publication of findings that have been, or will be, reported elsewhere and authors considering publication in such a journal should be aware of possible conflict with publication of the monograph version in Campbell Systematic Reviews. Publication in a journal after publication or in press status in Campbell Systematic Reviews should acknowledge the Campbell version and include a citation to it. Note that systematic reviews published in Campbell Systematic Reviews and co‐registered with the Cochrane Collaboration may have additional requirements or restrictions for co‐publication. Review authors accept responsibility for meeting any co‐publication requirements.

**I understand the commitment required to undertake a Campbell review, and agree to publish in the Campbell Library. Signed on behalf of the authors**:

**Table:** 

**Form completed by: Josef Novotný**	**Date: 16 Feb. 2018**
